# Promiximab-duocarmycin, a new CD56 antibody-drug conjugates, is highly efficacious in small cell lung cancer xenograft models

**DOI:** 10.18632/oncotarget.23708

**Published:** 2017-12-26

**Authors:** Lin Yu, Ying Lu, Yuqin Yao, Yu Liu, Yuxi Wang, Qinhuai Lai, Ruirui Zhang, Wenting Li, Ruixue Wang, Yuyin Fu, Yiran Tao, Shuli Yi, Lantu Gou, Ligong Chen, Jinliang Yang

**Affiliations:** ^1^ State Key Laboratory of Biotherapy and Cancer Center/Collaborative Innovation Center for Biotherapy, West China Hospital, West China Medical School, Sichuan University, Chengdu 610041, P.R. China; ^2^ Research Center for Occupational Respiratory Diseases, West China School of Public Health/No.4 West China Teaching Hospital, Sichuan University, Chengdu 610041, P.R. China; ^3^ Pharmacology & Pharmaceutical Sciences School of Medicine/Collaborative Innovation Center for Biotherapy, Tsinghua University, Beijing 100084, China

**Keywords:** CD56, small cell lung cancer, antibody-drug conjugates, duocarmycins

## Abstract

Small cell lung cancer (SCLC) is of a highly invasive and metastatic lung cancer subtype and there had not been effective targeted therapies. CD56, a cell surface marker highly expressed on most SCLC, is a promising therapeutic target for treatment of this aggressive cancer. In this study, we generated a novel anti-CD56 antibody named promiximab, characterized by high affinity, internalization and tumor specificity. Then, the promiximab was conjugated with a potent DNA alkylating agent duocarmycin via reduced interchain disulfides to yield the promiximab-Duocarmycin (promiximab-DUBA) conjugates. Mass spectrometry analysis showed promiximab-DUBA had an average DAR (Drug-to-Antibody Ratio) of about 2.04. *In vitro*, promiximab-DUBA exerted strong inhibitory effects on SCLC cell lines NCI-H526, NCI-H524 and NCI-H69, with IC50 values of 0.07 nmol/L, 0.18 nmol/L and 0.29 nmol/L, respectively. *In vivo* antitumor activity, promiximab-DUBA at the dose of 5 mg/kg and 10 mg/kg every three days with a total of three times were sufficient to induce sustained regression of NCI-H526 tumors over control treatment with promiximab. Mostly, no recurrence was observed until 65 days post treatment with promiximab-DUBA. In the NCI-H69 subcutaneous xenograft model, significant inhibition of tumor growth was also observed following administration of promiximab-DUBA at the dose of 5 mg/kg or 10 mg/kg. Moreover, body weight and histopathology of major organs (liver, spleen, heart, lung and kidney) showed no significant changes after treatment of promiximab-DUBA. In conclusion, promiximab-DUBA is highly efficacious in small cell lung cancer xenograft models, and provides a new immunotherapy approach for SCLC.

## INTRODUCTION

Small cell lung cancer (SCLC) is a much aggressive form of lung cancer that characterized by poor prognosis and rapid progress, which accounts for approximately 13-15% of all newly diagnosed lung cancer cases [1–3]. It is reported that the 5-year survival rate of SCLC patients remained low than 15% [4, 5]. Targeted therapies for non-small cell lung cancer (NSCLC) have been made great successes in the last decade [6]. However, few advances have been made in treatment of SCLC and novel approaches are urgently required [7, 8].

ADCs (Antibody Drug Conjugates), which are formed by monoclonal antibodies, linkers and chemotherapy drugs, have better targeting tumor effects but less toxic side effects than traditional chemotherapy drugs [9, 10]. Recent years, ADCs have been paid more attentions and there have been two drugs approved by the FDA (Food and Drug Administration) including Adctris® for systemic anaplastic large cell lymphoma (SALCL) [11] and Kadcyla® for HER2-positive breast cancer [12]. A few attempts to develop ADCs have been made to treat SCLC [13–15].

SC16LD6.5 (known as Rova-T), comprised of a humanized anti-DLL3 monoclonal antibody conjugated to a DNA-damaging pyrrolobenzodiazepine (PBD) dimer toxin has been entered clinical Phase I/II trials for SCLC [16, NCT01901653]. The other is Lorvotuzumab Mertansine (IMGN901), an anti-CD56 DM1 conjugates, consisting of a humanized CD56 antibody conjugated to microtubule inhibitor DM1 [17]. However, the phase II clinical trial of IMGN901 combination with etoposide/carboplatin (E/C) for extensive stage SCLC [NCT01237678] was experienced a higher risk of infection and one patient died, and it was not likely to significantly improve the rate of progression-free survival. As a result, the phase II clinical trial had been discontinued. One reason may be due to the inappropriate combination of three potent drugs, which could increase the toxicity and side effects. On the other hand, the three parts of ADCs, including monoclonal antibody, linker and chemotherapy drug need to be further optimized. Despite this, CD56, specially expressed on SCLC cell surface, is still of significance as ADCs target for SCLC [18–20].

Cisplatin or carboplatin based chemotherapies are the first-line treatments in SCLC, but it usually develops resistance to anti-cancer drugs and portends a dismal prognosis [4, 21–24]. It may be of certain significance to choose targeting DNA agents as payloads of ADCs for SCLC. Duocarmycins are DNA-alkylating agents with potent antitumor activity [25–27]. In a recent report, duocarmycin analogue had been chosen as payloads for a HER2-targeting antibody-drug conjugates SYD985, showing potent antitumor effects [28].

In this research, we designed a novel CD56-targeted ADC (promiximab-DUBA), conjugating of an anti-CD56 hIgG1 antibody with a linker-drug based on duocarmycins by reduced interchain disulfides. The promiximab-DUBA was characterized by specific binding capacity, high affinity and effective internalization. It is highly efficacious in small cell lung cancer *in vitro* and *in vivo*, providing a new immunotherapy approach for SCLC.

## RESULTS

### Promiximab was a suitable antibody for antibody-drug conjugates

**Figure 1:**
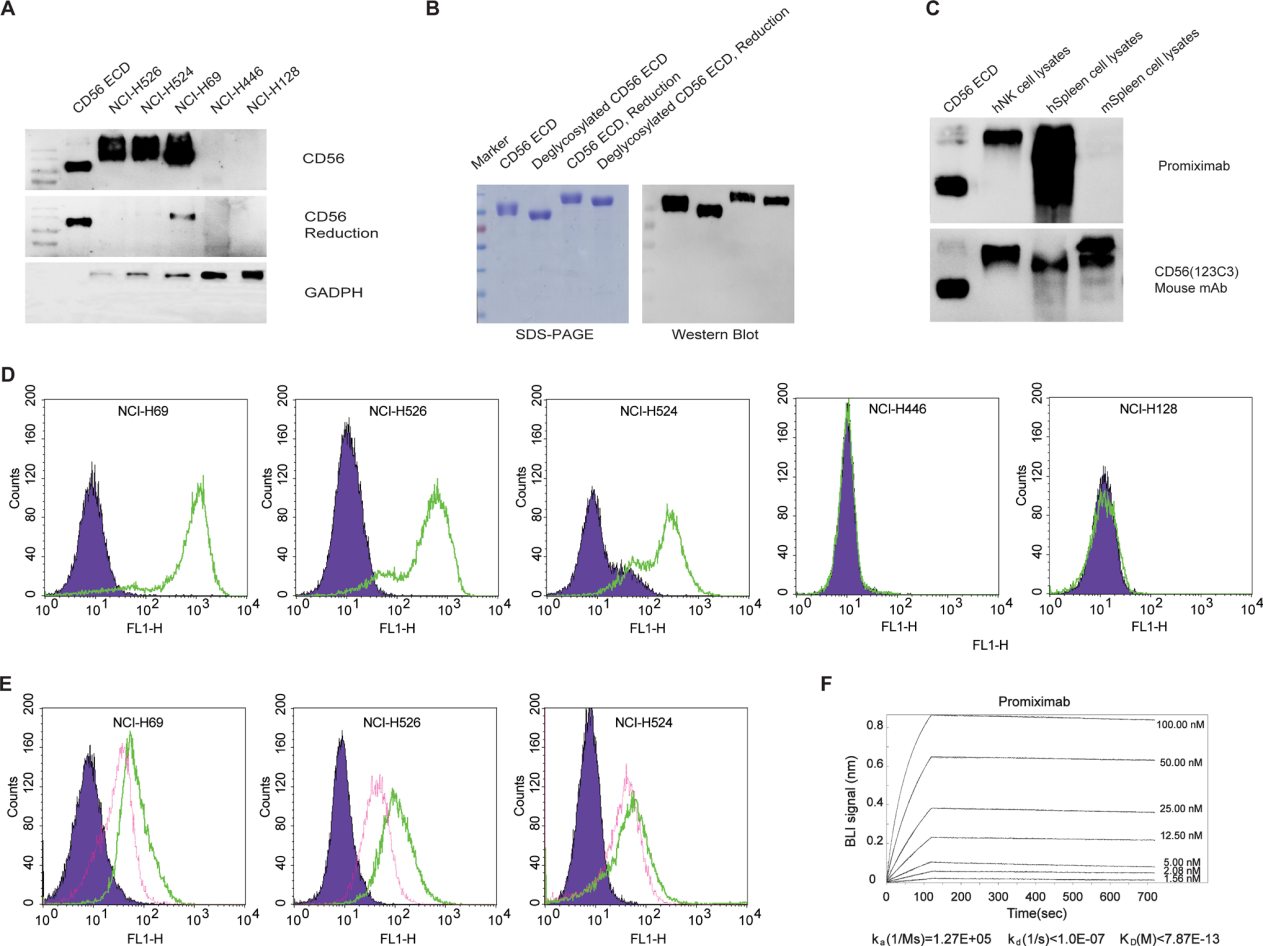
Specificity, internalization and affinity of promiximab **(A)** Western blot detected CD56 expression in small cell lung cancer cell lines cell lysates including CD56 extracellular domain (CD56 ECD), NCI-H526, NCI-H524, NCI-H69, NCI-H128, NCI-H446 using promiximab antibody. **(B)** Western blot was required for detection of CD56 extracellular domain on the condition of deglycosylation or non-deglycosylation using promiximab antibody. **(C)** Identification of cross reactivity of chimeric antibody with human NK (hNK) cell lysates, human spleen (hSpleen) cell lysates, mouse spleen (mSpleen) cell lysates by western blot, CD56 ECD as control. **(D)** Binding capacity of promiximab to small cell lung cancer cell lines were detected by flow cytometric analyses and SCLC cell lines incubated with PBS (purple), Promiximab (green), respectively. **(E)** Internalization of promiximab in CD56-positive small cell lung cancer cell lines were also assessed by flow cytometric analyses, cell lines were incubation with PBS (purple, 4°C, 3 h), promiximab (green, 4°C, 3 h), promiximab (fuchsia, 37°C, 3 h). **(F)** Biolayer interferometry binding assay were used for monitoring the interaction kinetics between CD56 ECD and different concentrations of promiximab as described in Materials and Methods. Three independent experiments were conducted and similar results were obtained.

Through hybridoma and humanization technology, we generated the new anti-CD56 antibody promiximab and it was characterized by the following properties. Promiximab can bind to the CD56 expressed on the surface of NCI-H526, NCI-H524 and NCI-H69 SCLC cells. However, there were almost no bindings to the NCI-H128, NCI-H446 SCLC cell lines which may indicate the spatial specificity binding of promiximab (Figure 1A and 1D) [29]. Furthermore, it was bound to the deglycosylated CD56 ECD (Figure 1B). For the immunological cross-reactivity, promiximab reacted with CD56 expressed in human NK cells and human spleen, but low signal was detected promiximab with spleen cell lysates from Balb/C by Western blot (Figure 1C). For ADCs, high binding affinity and internalization of the antibodies are of importance. Biolayer interferometry analysis showed promiximab with a high binding affinity 0.78 pmol (k_a_ (1/Ms) = 1.27E+05, k_d_ (1/s) < 1.0E-07, K_D_ < 7.8E-13) for CD56 extracellular antigen (Figure 1E). The internalization efficiencies of promiximab in NCI-H526, NCI-H524 and NCI-H69 cell lines were separately 68.19%, 53.14% and 64.97%, detected by flow cytometry. Taken together, the novel CD56 antibody promiximab with specific binding capacity, high affinity and effective internalization is suitable for using in an effective ADCs.

### Promiximab-DUBA conjugates were prepared via reduced interchain disulfides

**Figure 2:**
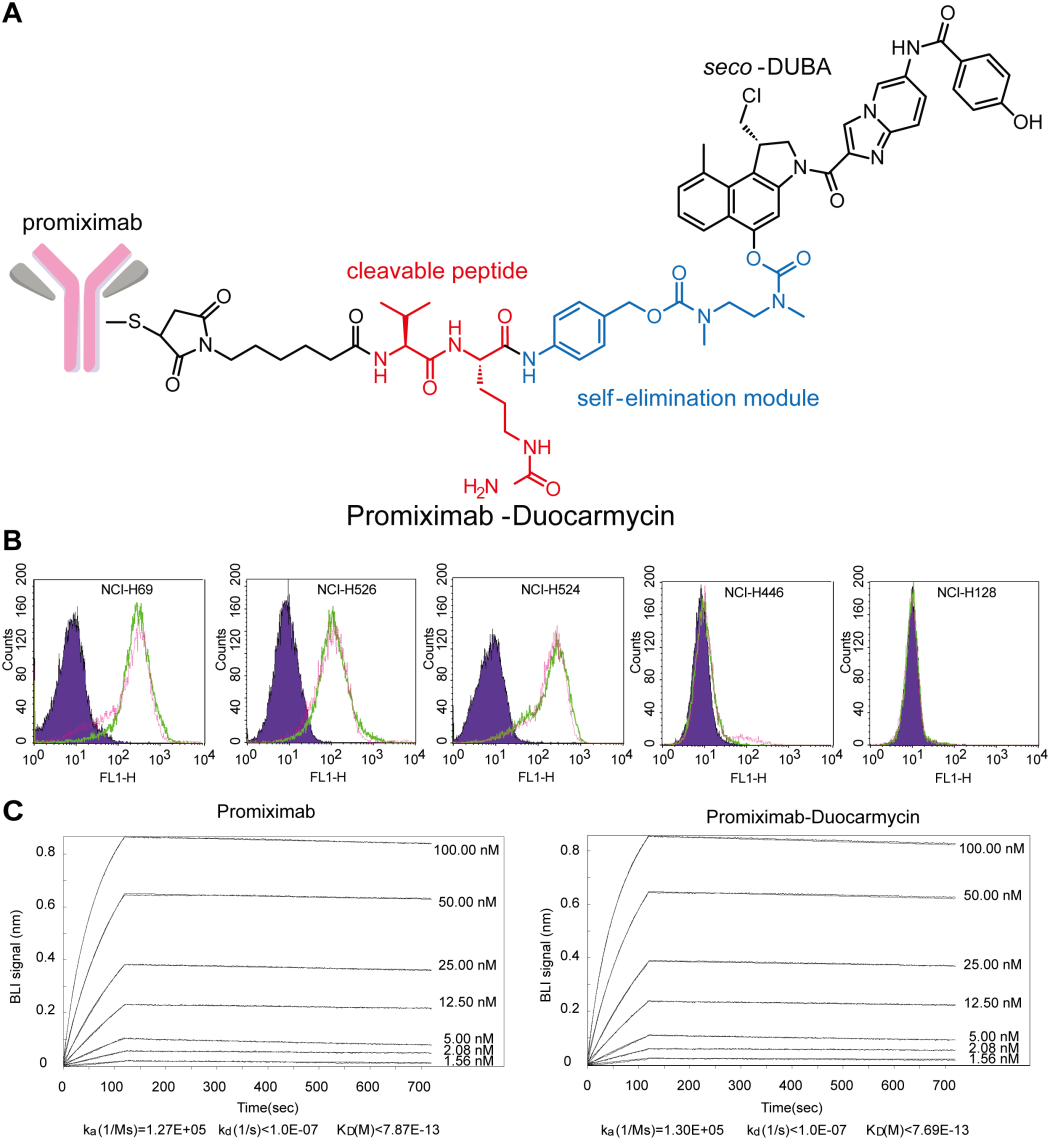
Structure, binding capacity, and affinity kinetics of promiximab-DUBA **(A)** The scheme of the promiximab-DUBA including the antibody, the linker, and the drug. **(B)** Binding capacity of promiximab after conjugated with linker payload. Binding capacity of promiximab to small cell lung cancer cell lines were detected by flow cytometric analyses and small cell lung cancer cell lines incubated with PBS (purple), promiximab (green), promiximab-DUBA (fuchsia) respectively. **(C)** Biolayer interferometry binding assay were used for monitoring the interaction kinetics between CD56 extracellular domain and different concentrations of promiximab-DUBA as described in Materials and Methods.

Promiximab-DUBA was generated by conjugating linker-duocarmycins to promiximab and its scheme is shown in Figure 2A. The average DAR of the conjugates was about 2.04 as determined by mass spectrometry analysis. To investigate the influence on the binding capacity of antibody after conjugating linker-duocarmycins, SCLC cell lines were incubated with promiximab-DUBA orpromiximab. Three independent results all showed that promiximab-DUBA and promiximab bound to CD56-expressing cells in a similar manner, which indicated conjugating promiximab with duocarmycins, did not influence the binding capacity of promiximab (Figure 2B). Biolayer interferometry analysis demonstrated that promiximab and promiximab-DUBA had a similar binding kinetics with CD56 extracellular antigen (Figure 2C). These results indicated that there were no change in binding capacity and affinity of promiximab after conjugated linker-duocarmycins.

### Promiximab-DUBA exerted potent *in vitro* tumor cell killing activity

**Figure 3:**
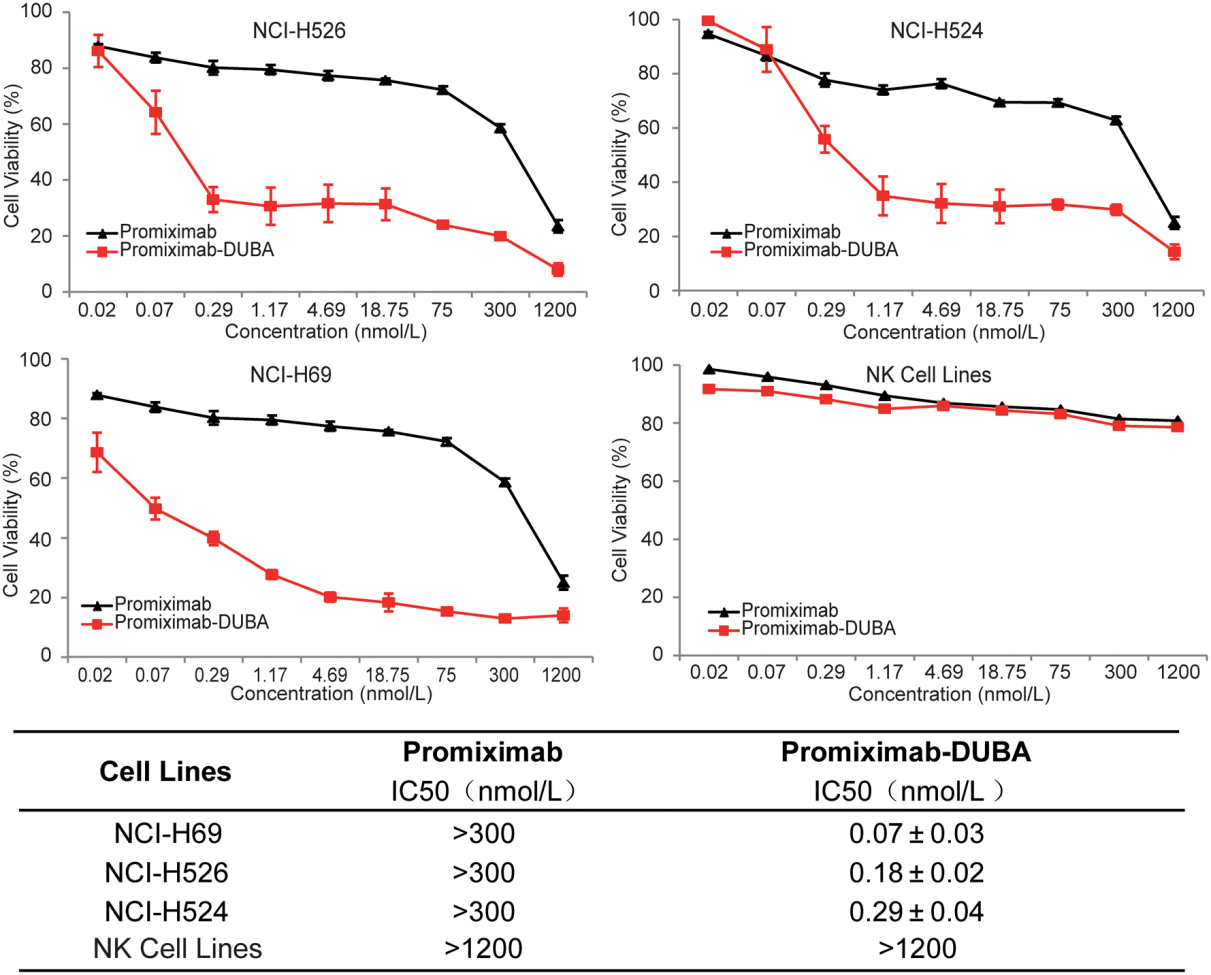
*In vitro* cytotoxicity assay of promiximab and promiximab-DUBA Small cell lung cancer cell lines and human NK cells were treated with various concentrations of promiximab and promiximab-DUBA for 72 h. Untreated cells were used as controls. The IC50 values obtained after a 72 h cell exposure were summarized in Table. Cell viability was determined by three independent experiments of CCK-8 assay and calculated as the absorbance ratio of treated to control groups. Data were presented as Mean±SEM.

The anti-proliferative activities of promiximab-DUBA and promiximab were exposed to SCLC cell lines and human NK cells at concentrations ranging from 0.02 to 1200 nmol/L for 72 hours. The results showed promiximab-DUBA exerted strong inhibitory effects in NCI-H526, NCI-H524 and NCI-H69 cells, with IC50 values of 0.07 nmol/L, 0.18 nmol/L and 0.29 nmol/L, respectively. Even though promiximab can bind to CD56 expressed on NK cells surface, tumor cells have been demonstrated to 60 times higher levels of CD56 than that of NK cells [30]. Consistent with the literature, we found that the inhibitory effects of promiximab-DUBA to human NK cells were less effective with IC50 values higher than 1200 nmol/L (Figure 3). From these results, we can learn promiximab-DUBA exhibited a potent tumor-killing activity on SCLC cells *in vitro*, ^*^p<0.05.

### Promiximab-DUBA was highly efficacious in small cell lung cancer xenograft models

**Figure 4:**
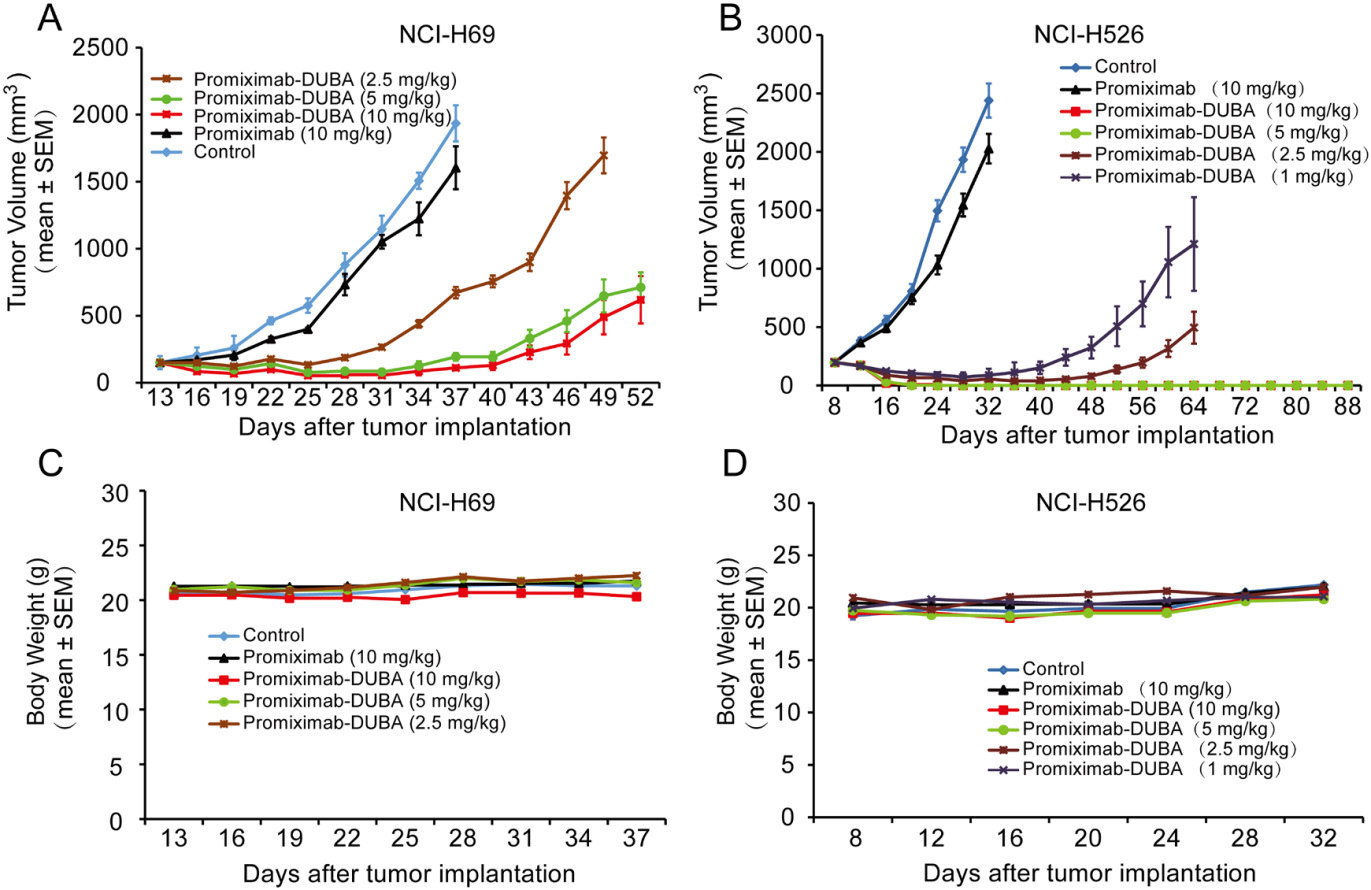
Therapeutic effects of promiximab and promiximab-DUBA against NCI-H526 and NCI-H69 small cell lung cancer xenografts Subcutaneous tumor-bearing mice were treated with buffer, promiximab and promiximab-DUBA at 1 mg/kg, 2.5 mg/kg, 5 mg/kg and 10 mg/kg every three days for three times. **(A)** Promiximab-DUBA showed more powerful inhibition of NCI-H526 subcutaneous xenografts growth compared with promiximab, even at lower dose. **(B)** Body weight of mice did not show significant changes during treatment for subcutaneous xenografts model. One out of two independent tumor progression experiments of the subcutaneous xenograft models had been shown. Data were presented as Mean±SEM.

Subcutaneous CD56-expressing NCI-H69 and NCI-H526 xenograft models were used for evaluating the anti-tumor efficacies of promiximab-DUBA. In the NCI-H69 subcutaneous xenograft model, two out of five mice showed complete remission without regrowth for the promiximab-DUBA (10 mg/kg) treatment group, whereas one out of five showed complete remission without regrowth for the promiximab-DUBA (2.5 and 5 mg/kg) treatment group (Figure 4A). For the NCI-H526 xenograft model, complete tumor regressions were observed in mice receiving 10 mg/kg and 5 mg/kg promiximab-DUBA and no regrowth were observed in a long term after termination of the treatment. For the promiximab-DUBA (2.5 mg/kg and 1 mg/kg) treatment groups, significant inhibitions of tumor growth were also observed (Figure 4B). Besides, the body weight of those mice group kept increasing after cessation of treatment (Figure 4C-4D). All tumor progression experiments have independently repeated twice and obtained similar results, and a typical result had been shown. These data indicated excellent anticancer activities of promiximab-DUBA in the subcutaneous xenograft models.

### Promiximab-DUBA did not significantly influence on the histopathology of major organs of treated mice

**Figure 5:**
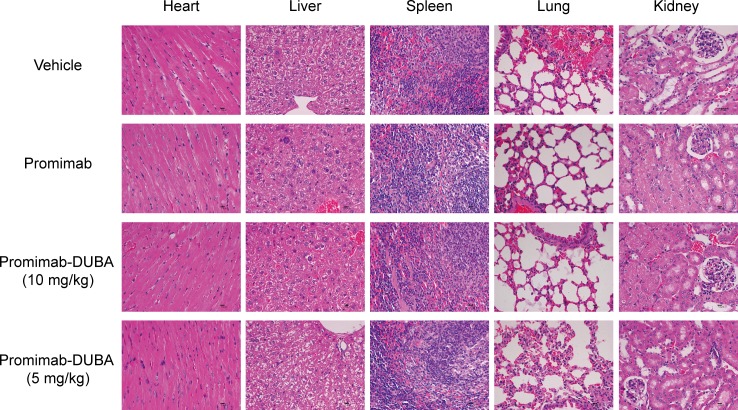
Histological and pathological observations for mice received multi-dose administration of promiximab or promiximab-DUBA Mice were injected with vehicle, 5, and 10 mg/kg of promiximab or promiximab-DUBA. Tissue samples of heart, liver, spleen, lung, and kidney were collected on the day 20 after the last injection. The representative images of heart, liver, spleen, lung, and kidney revealed no visible side toxicities.

H&E analysis of major organs from Balb/c mice after was chosen to examine potential side toxicities of promiximab-DUBA. Main organs including heart, liver, spleen, lung and kidney were harvested at day 20 after the last intravenous injection. As observed (Figure 5), no obvious changes were observed after the administration of promiximab-DUBA, compared with the normal organ tissues from mice receiving vehicle.

## DISCUSSION

Therapeutic concept of ADCs is to use an antibody to deliver a cytotoxic drug selectively targeted to the tumor tissue expressing an antigen on the surface of malignant cells [31]. In this study, we described a novel anti-CD56 ADCs, promiximab-DUBA, an anti-CD56 hIgG1 antibody conjugated with a new linker-drug based on duocarmycins by reduced interchain disulfides.

ADCs combine the selectivity of a monoclonal antibody with the killing potency of a cytotoxic drug [32, 33]. The design of a successful anti-CD56 ADCs required a careful balance between efficacy and safety and several factors were taken into consideration. Firstly, an antibody plays an important role in a successful ADC and exploring different antibodies against CD56 still make sense. Secondly, the cytotoxic payload is a critical component of an ADC and should be chosen carefully. Thirdly, the ADCs used in clinical have been manufactured through cysteine disulfide bond or lysine-based conjugations, producing mixtures of ADC species [11, 12]. In this study, we prepared the new antibody promiximab with binding capacity, high affinity and effective internalization which may be suitable for anti-CD56 ADCs. And the potent DNA alkylating agent duocarmycin was selected as a payload to explore whether DNA inhibitors could be effective payload. As we known that IMGN901 was generated by lysine-based conjugations at the average modification level of 3.7 linker-DM1 moieties per antibody molecule [12, 30]. In our studies, the promiximab-DUBA was produced by conjugating the antibody promiximab with linker-duocarmycins via reduced interchain disulfides conjugation method at the average DAR of 2.04. Taken together, the new prepared ADCs in this study was different from IMGN901 in terms of antibody, payload and conjugation method. Most importantly, promiximab-DUBA showed potent anti-tumor activity *in vitro* and *in vivo*.

The anti-proliferative activities of promiximab-DUBA on CD56-expressing SCLC cell lines were potential effect with IC50 in the nanomolar range. Although CD56 is biomarker for NK cells, promiximab-DUBA was not effective with IC50 higher than 1200 nmol/L because of the low expressing of CD56 on the surface of NK cells compare to tumor cells (Figure 3) [30]. Promiximab-DUBA was also highly efficacious *in vivo*, with tumor regression generally achieved with three times injection at a dose of 5 mg/kg and 10 mg/kg in the NCI-H526 xenograft model. Besides, the body weight of mice from the promiximab-DUBA group kept increasing after cessation of treatment and H&E staining showed no obvious systemic toxicity risk after the administration of promiximab-DUBA.

The current studies suggest that promiximab-DUBA are very potent in NCI-H526 and NCI-H69 SCLC xenograft models and worth further exploration. In addition, CD56 as a target is of significance for SCLC therapy. These promising results provide valuable references for the future studies on SCLC immunotherapy.

## MATERIALS AND METHODS

### Cell lines and reagents

SCLC cell lines NCI-H128, NCI-H446, NCI-H524, NCI-H526 and NCI-H69 were obtained from American Type Culture Collection. Cells were cultured in RPMI-1640 supplemented with 20% FBS, penicillin (100 U/mL) and streptomycin (100 μg/mL). Natural killer (NK) cells were given by Dr. Liang. All cells were maintained in a humidified 5% CO2 atmosphere at 37°C. The duocarmycin-linkers were synthesized by State Key Laboratory of Biotherapy and Cancer Center/Collaborative Innovation Center for Biotherapy.

### Generation of the promiximab

First, the CD56 antibody was derived from using a C-terminal 6×His tag recombinant CD56 extracellular domain (CD56 ECD). Second, the fusions B cells of the immunized Balb/c mice with myeloma cells were carried out to the production of hybridomas. Third, the resulting monoclonal populations of cells were screened by multiple rounds of limiting serial dilutions and by the analysis of the antibodies secreted into the culture supernatants using enzyme-linked immunosorbent assay (ELISA), western blotting and flow cytometry [34–36]. After sequencing variable regions of hybridoma cell lines, the candidate antibody was humanized by CDR (Complementarity-Determining Region) grafting and cloned into human IgG1/k constant domains to create the parental antibody, named promiximab. The DNA encoding this antibody was cloned into a pTT5 vector, an in-mammalian cell expression plasmid, transiently expressed in HEK293 cells, and then purified with HiTrap™ rProtein A FF columns (GE Healthcare) [37, 38].

### Preparation of promiximab-DUBA

The antibody concentration was adjusted to 5 mg/mL in buffer containing 20 mmol/L phosphate buffer, 20 mmol/L NaCl and 1 mmol/L diethylenetriaminepentaacetic acid (DTPA, #D6518-5G, Sigma-Aldrich), pH7.0, and mixed with 3-fold molar excess over antibody tris(2-carboxyethyl)-phosphine (TCEP, #C4706-2G, Sigma-Aldrich) at 37°C for 3 hours. The thiol concentration in the partially reduced promiximab was determined by 5, 5-dithiobis (2-nitrobenzoic acid) (DTNB, D0944, TCI) and an average of approximately three disulfide bonds were reduced. The duocarmycin-linker solution to be used in the conjugations was prepared by diluting drug-linker from N, N-dimethylacetamide (DMA) stock solution. The volume of drug-linker solution was calculated to contain 6-mol drug-linker/1 mol antibody and the conjugation reaction was incubated with gentle shaking at room temperature for 3 hours. The conjugates were buffer-exchanged into 20 mmol/L phosphate buffer/50 mmol/L NaCl (pH 7.0) by HiTrap Desalting. The final product drug-antibody ratio (DAR) was determined by size exclusion chromatography (SEC) and mass spectrometry analysis [28, 39–42].

### Flow cytometry

SCLC cells (1×10^6^ cells/tube) were centrifuged and suspended in 100 μL PBS (pH 7.0). Then the cells were incubated with a concentration of 1μg/ml of promiximab, ADCs or PBS for 30 min at 4°C, respectively. After incubation, cells were washed three times with PBS and then labeled with FITC-conjugated AffiniPure goat anti-human IgG (Jackson ImmunoResearch) about 30 min at 4°C. After washing three times with PBS, the fluorescence intensity of FITC was determined using flow cytometer (BD FACSCalibur) [43]. Three independent experiments of flow cytometric analysis were conducted.

### Western blot

SDS-PAGE and western blot analyses were performed as standard procedures [44]. Total proteins of SCLC cell lines were extracted by a RIPA lysis buffer (#P0013B, Beyotime), supplemented with protease inhibitor cocktail diluted at 1:100 (Thermo Scientific). All samples were separately prepared in both reduced and denatured condition and native and non-reduced condition. For the deglycosylation, CD56 ECD (40 μg) was incubated with 0.6 uL of PNGase F (500 U /μL, #P0704, NEB) for 4 hours at 37°C in 50 mM Tris, pH 7.0. Primary antibody (promiximab) was diluted in 5% BSA in TBST with the final concentration of 1 μg/mL. And the signals were detected by an enhanced chemiluminescence detection kit (Millipore, USA) and images were captured by the ImageQuant LAS 4000 mini system (GE, USA).

### Internalization

CD56-expressing SCLC cell lines (1×10^7^ cells/tube) were centrifuged and resuspended in PBS (pH 7.0). Then the cells were incubated with a concentration of 10 μg/ml of promiximab, ADCs or PBS for 30 min at 4°C, respectively. These cells were split into two groups after a wash step with ice-cold 1x PBS. For one part of the cells, internalization was assessed upon incubation at 37°C, 3h (500 μL cell solution/vial). The other part was used as control cells for the total cell surface binding and was incubated at 4°C. After the indicated incubation times, cells were washed three times with PBS and then labeled with 50 μL of FITC-conjugated AffiniPure goat anti-human IgG (Jackson ImmunoResearch) about 30 min at 4°C. Fluorescence intensities were determined by flow cytometry (BD FACSVerse, Fanklin Lakes, NJ) and indicated as the median fluorescence intensity (MFI). Percentage internalization was calculated by dividing the ‘MFI of surface internalized subtracted for untreated cells’ by the ‘MFI of total bound subtracted for untreated cells’ multiplied with 100 [45]. Three independent experiments of flow cytometric analysis were conducted.

### Affinity assay

All interaction experiments were conducted at 25°C in PBST (20 mmol/L phosphate buffer, 0.05% tween 20, 500 mmol/L NaCl, pH 7.4) using an Octet Red 96 instrument (Fortebio). The final volume for all solutions was 200 μL. Biolayer interferometry binding analyses were performed according to standard procedures. Firstly, different concentrations of promiximab-DUBA or promiximab were loaded onto biosensor (Protein A, ForteBio) for 2 min. Secondly, recombinant CD56 ECD was loaded to association with the antibody or ADC for 3 min. Finally, dissociation between promiximab-DUBA or promiximab and CD56 antigen were measured for 16 min. The real-time data were analyzed using Biaevaluation 4.1 (GE Healthcare). Association and dissociation profiles, as well as steady-state equilibrium concentration curves, were fitted using a 1:2 binding model [46–48].

### *In vitro* cytotoxicity

The growth inhibition of the promiximab-DUBA conjugates and promiximab on small cell lung cancer cells were determined by the cell counting kit-8 (Dojindo Laboratorie, CK04) assay. Cells (1.2×10^3^/well-1×10^4^/well) were seeded in 100 μL medium per well in 96-well plates. After 24 h cultured, cells were treated with promiximab or promiximab-DUBA of various concentrations in culture medium, and culture medium was used as control. After 72 h exposure, a volume of 20 μL CCK-8 solutions was added to each well. After 1.5 h incubation, the plates were agitated on a shaker for 5 min and the absorbance at 450 nm was measured by ELISA reader (Thermo, Multiskcan MK3). All experiments were conducted independently in triplicate. The effects of each agent on the proliferation of ovarian cancer cell lines were expressed as the % cell growth inhibition using the following formula: % inhibition= [(A450 of control-A450 of treated cells)/A450 of control] ×100%. The 50% inhibiting concentration (IC50) was calculated by SPSS software version 17.0 [49]. Statistical analysis: The significant difference ADCs and antibody treated cells was statistically analyzed by paired Student's t test, and results were considered statistically significant when P < 0.05.

### *In vivo* antitumor activity

Female Balb/c nude mice (5-6 weeks old) were purchased from Beijing HFK Bioscience and were acclimated for one week before the experiment. Animal experiments were approved by the Institutional Animal Care and Treatment Committee of State Key Laboratory of Biotherapy in Sichuan University. All *in vivo* experiments of study the antitumor effect of the promiximab-DUBA conjugate have independently repeated twice. To establish subcutaneous CD56-expressing NCI-H526 and NCI-H69 xenograft model, the mice were respectively given a single subcutaneous injection supplemented with Matrigel™ Basement Membrane Matrix (BD Bioscience) with about 2×10^7^ NCI-H526 or NCI-H69 cells in 150 μL cell culture medium (RPM 1640) [50]. One or two weeks later until the tumor sizes reached about 150-200 mm^3^, the mice divided into four groups, and each group has 6-7 mice. Promiximab-DUBA (1, 2.5, 5, 10 mg/kg), promiximab (10 mg/kg) and a control (vehicle) were via tail vein injection to mice every three days, with a total of three times. For the subcutaneous xenograft model, tumor volume and body weight were monitored every three days throughout the treatment. When the subcutaneous xenografts over 2000 mm^3^, the mice were euthanized and the experiment was terminated.

### H&E staining

Tissue samples of heart, liver, spleen, lung and kidney from tumor-bearing mice treated with buffer, promiximab and promiximab-DUBA were fixed in 4% phosphate-buffered formaldehyde and paraffin-embedded as conventional methods. For histopathological analysis, tissue sections were stained with hematoxylin and eosin (H&E). All systemic tissue toxicity was performed at the University of Miami's Department of Pathology. Histopathological evaluations were performed in accordance with the guidelines of the Society of Toxicologic Pathology [51]. All images were captured using digital trinocular camera microscope and analyzed by software Image-Pro Plus 6.0 (Media Cybernetics, Inc., USA), and the repeated experiments showed similar results which were statistically significant.

## Abbreviations

SCLC: Small cell lung cancer
ADC: antibody drug conjugates
DAR: drug-antibody ratio
CD56 ECD: CD56 extracellular domain
hNK: human natural killer
hSpleen: human spleen
hSpleen: mouse spleen
